# Heading toward Miniature Sensors: Electrical Conductance of Linearly Assembled Gold Nanorods

**DOI:** 10.3390/nano13091466

**Published:** 2023-04-25

**Authors:** Marisa Hoffmann, Christine Alexandra Schedel, Martin Mayer, Christian Rossner, Marcus Scheele, Andreas Fery

**Affiliations:** 1 Leibniz-Institut für Polymerforschung Dresden e.V., Institute of Physical Chemistry and Polymer Physics, Hohe Str. 6, 01069 Dresden, Germany; 2 Physical Chemistry of Polymeric Materials, Technische Universität Dresden, Bergstr. 66, 01069 Dresden, Germany; 3 Center for Advancing Electronics Dresden, Technische Universität Dresden, Helmholtzstr. 18, 01069 Dresden, Germany; 4 Institute of Physical and Theoretical Chemistry, Eberhard Karls Universität Tübingen, Auf der Morgenstelle 18, 72076 Tübingen, Germany; 5 Dresden Center for Intelligent Materials (DCIM), Technische Universität Dresden, 01069 Dresden, Germany

**Keywords:** self-assembly, gold nanorods, anisotropic conductance

## Abstract

Metal nanoparticles are increasingly used as key elements in the fabrication and processing of advanced electronic systems and devices. For future device integration, their charge transport properties are essential. This has been exploited, e.g., in the development of gold-nanoparticle-based conductive inks and chemiresistive sensors. Colloidal wires and metal nanoparticle lines can also be used as interconnection structures to build directional electrical circuits, e.g., for signal transduction. Our scalable bottom-up, template-assisted self-assembly creates gold-nanorod (AuNR) lines that feature comparably small widths, as well as good conductivity. However, the bottom-up approach poses the question about the consistency of charge transport properties between individual lines, as this approach leads to heterogeneities among those lines with regard to AuNR orientation, as well as line defects. Therefore, we test the conductance of the AuNR lines and identify requirements for a reliable performance. We reveal that multiple parallel AuNR lines (>11) are necessary to achieve predictable conductivity properties, defining the level of miniaturization possible in such a setup. With this system, even an active area of only 16 µm^2^ shows a higher conductance (~10^−5^ S) than a monolayer of gold nanospheres with dithiolated-conjugated ligands and additionally features the advantage of anisotropic conductance.

## 1. Introduction

Metal nanoparticles (NPs) are increasingly used as key elements in the fabrication and processing of advanced electronic systems and devices. At a comparably small size (e.g., >1.4 nm for gold [1]), an electronic band structure develops, and metal NPs become electrically conductive. In addition, gold NPs can serve as model systems in fundamental research [2] because of their precise shapes, chemical stability, ease of surface functionalization and processability.

In many applications, the conductivity of gold NP assemblies is crucial. It has been exploited, e.g., in the development of gold-NP-based conductive inks [3]. Assembled metal NPs and metal NP films can also be implemented into strain-sensitive [4] or resistive pressure-sensitive devices [5,6,7], which can be used, e.g., in healthcare [8]. Moreover, the fact that gold NPs possess a high surface-to-volume ratio proved them useful as sensing platforms to detect alcohols or neurotransmitters [9,10], solvent vapor [11,12,13], or electrochemical reactions [14] upon adsorption. Furthermore, arrays of parallel colloidal nanowires can be implemented to create surfaces with anisotropic conductance [15]. These could be used as interconnection structures to build electrical circuits, e.g., for signal transduction of directional mechanical events.

The step from single NPs to electronic components such as colloidal nanowires requires NP arrangement into tailored supracolloidal structures. The assembly of metal NPs into lines, as well as the optical properties of the resulting colloidal wires have been the subject of a plethora of investigations. Lines of plasmonic nanoparticles show optical effects, such as the coupling of plasmonic modes [16,17,18,19], and higher enhancement factors for surface-enhanced Raman spectroscopy (SERS) than single plasmonic NPs [20,21]. Whereas top-down methods can fabricate metal nanowires of arbitrary shape, large-scale fabrication is challenging, and lithographic methods are energy consuming and environmentally critical. In contrast, colloidal nanowire fabrication via self-assembly is scalable and has a reduced environmental footprint. Various bottom-up methods have been employed to assemble metal NPs into lines and stripes, including spin coating [20,22,23], dip coating [19,24], microfluidics [25] and capillary-assisted assembly [26]. The above-mentioned papers partially include testing the conductivity of the fabricated linearly assembled NPs. Studies on the conductivity mechanisms in three-dimensional networks of metal NPs [27] and in non-linear gold nanosphere chains [28] report that hopping dominates the charge transport at room temperature.

However, even for the fabrication of colloidal gold nanowires, top-down methods [17,26] are often used. Among the existing colloidal nanowires, there are only a few examples of linear gold nanorod (AuNR) assemblies. AuNR lines offer lower percolation thresholds for electrical conduction compared to colloidal nanowires composed of less anisotropic nanoparticles [29]. Therefore, they are well-suited for creating micron-sized surfaces with anisotropic conductivity. Most of the fabricated AuNR lines have widths in the (sub-)micrometer range, which is unfavorable for pushing the limit of device miniaturization. The printed stripes of poly [2-(3-thienyl)-ethyl-oxy-4-butylsulfonate]-functionalized AuNRs by Reiser et al. had low resistivities of 10^−6^ Ωm, but, on the other hand, widths of several hundred micrometers [3]. Rey et al. used a procedure of template-assisted capillary assembly of AuNRs by polydimethyl siloxane (PDMS) templates based on electron-beam lithography-made masters, which relies on lithographic processing steps. The resulting single AuNR lines had gaps of 5–7 nm between the AuNR tips and did not show any measurable conductance [26]. Despite the partial use of expensive methods, in none of these examples were the AuNR lines obtained with small dimensions while still maintaining acceptable conductivity values.

In contrast, our bottom-up, template-assisted, self-assembled AuNR lines feature comparably small widths [3,30] and better conductivities than other comparable assemblies, such as gold nanospheres with dithiolated-conjugated ligands [31]. Nevertheless, the bottom-up approach for preparing AuNR lines poses the question about the consistency of charge transport properties between individual lines, as this approach leads to heterogeneities among those lines with regard to AuNR orientation, as well as line defects. However, consistent charge transport properties are essential for future device integration. Therefore, the motivation of this work was to test the conductance of the AuNR lines and identify requirements for reliable performance. We revealed that multiple parallel AuNR lines (>11) are necessary to achieve predictable conductivity properties, defining the level of miniaturization possible in such a setup. Thereby, we set the foundation to use AuNR lines as resistance-based sensor wires or as anisotropically conducting surfaces in devices on the meso scale.

## 2. Materials and Methods

**Materials.** Cetyltrimethylammonium bromide (CTAB, 99%) was received from Merck chemicals. Cetyltrimethylammonium chloride (CTAC), HAuCl_4_·3H_2_O (99.9%), HBr (48% in water), silver nitrate (AgNO_3_, 99.9999%), sodium borohydride (NaBH_4_, 99%), and hydroquinone (99%) were purchased from Sigma-Aldrich. Photoresist maP-1215 and the developer maD-331/S were purchased from micro-resist technology. All chemicals were used as received. Purified water (Milli-Q-grade, 18.2 MΩ cm at 25 °C) was used as obtained from the purification system.

**Gold Nanorod (AuNR) Synthesis.** AuNRs were synthesized with minor modifications, as published elsewhere [32]. Briefly, seed particles were prepared by adding 3 mL of a freshly prepared 0.01 M NaBH_4_ solution in a 47 mL mixture of 0.1 M CTAB and 0.25 mM HAuCl_4_ under vigorous stirring at 40 °C. The solution was stirred rapidly for 2 min, followed by continued slow stirring at 32 °C for 30 min. A 1 L of 0.1 M CTAB solution was prepared and 5 mL of 0.1 M HAuCl_4_ solution (fc.: 0.5 mM), 500 µL of 0.1 M HBr, and 4 mL of 0.1 M AgNO_3_ were added (f.c.: 0.4 mM). Of the 0.1 M hydroquinone solution (f.c.: 5 mM), 50 mL was added as the reducing agent while stirring and 2 min later, 18 mL of the as-prepared seed solution was added and kept at 32 °C for at least 48 h.

**Figure 1:**
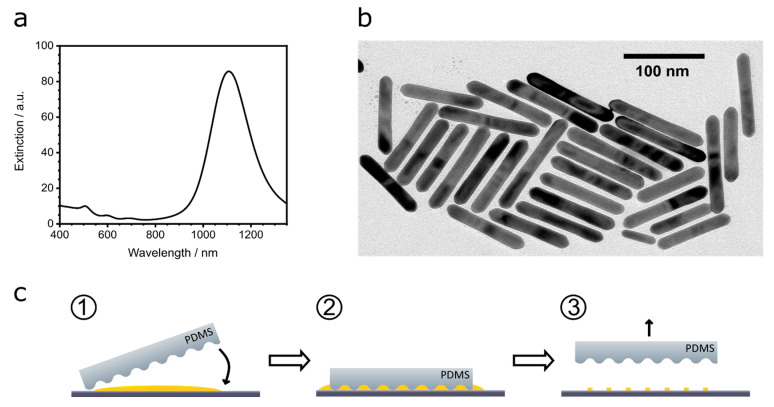
(**a**) Vis-NIR spectrum of the AuNRs with the transversal plasmon mode at 505 nm and the longitudinal plasmon mode at 1107 nm. (**b**) TEM image of AuNRs and (**c**) scheme of the template-assisted self-assembly of the AuNRs from aqueous solution on a Si/SiO_2_ (230 nm) wafer with a wrinkled PDMS template.

**Vis-NIR measurements.** The extinction spectrum of the CTAC-stabilized AuNRs in aqueous solution were acquired with the spectrophotometer Cary 5000 (Agilent Technologies Deutschland GmbH, Germany). With the intensity of the extinction spectrum (Figure 1) at a wavelength of 400 nm (interband transition of gold [33], the concentration of the AuNR dispersion was calculated.

**TEM measurements.** Transmission electron microscopy (TEM) measurements were performed with a Libra200 (Zeiss, Germany) operated at an acceleration voltage of 200 kV. For TEM analysis, 1 mL as-synthesized nanoparticle solutions were concentrated to 50 μL via centrifugation, and washed twice to reduce the surfactant concentration below the critical micelle concentration (cmc; ~0.9 mM). Subsequently, 2–5 μL of these solutions were dried on a 400 mesh copper grid covered with a carbon support film. The geometric dimensions of over 250 AuNRs were determined by using ImageJ.

**Template Fabrication.** Wrinkled PDMS templates were fabricated as follows. Prepolymer and agent from the PDMS Sylgard 184 kit were mixed in a ratio of 10:1. It was hardened for 1 day at room temperature and subsequently cured at 80 °C for about 4 h. Stripes of 1.0 cm × 4.5 cm were cut from the cooled PDMS. To create wrinkles on the PDMS surface, a PDMS stripe was formed to 140% of its original length by a custom-made stretching device. After treatment with oxygen plasma (80 W, 0.2 mbar) for 5 min, the PDMS stripe was released to its original length. This procedure resulted in wrinkles with a wavelength of about 950 nm and a depth of ca. 220 nm.

**Substrate Fabrication.** Si/SiO_2_(230 nm) wafers (15 × 15 mm) were used as the substrate, and gold electrodes of 80 µm width and 1.5 µm channel length (finger width 10 µm) were photolithographically prepared using a chromium adhesion layer. Photoresist maP-1215 was spin-coated on the wafer (3000 rpm; 30 s) and soft-baked on the hot plate (100 °C) for 60 s. A Karl Süss Mask Aligner MA/BA6 was used for light exposure and after development in maD-331/S, the substrates were metallized with 3 nm chromium and 30 nm gold in a PLS570 evaporator. The lift-off was done in acetone in an ultrasonic bath for two minutes.

**Figure 2:**
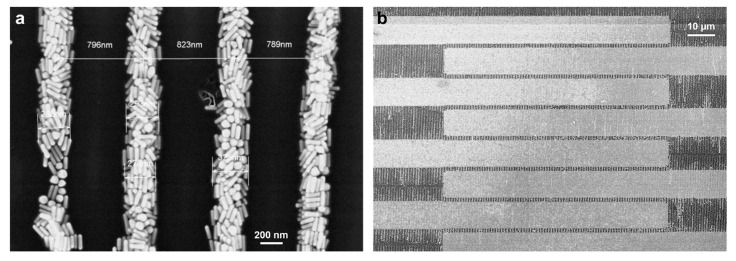
SEM images of (**a**) AuNR lines on Si/SiO_2_ wafer and (**b**) on an FET substrate, with the AuNR lines perpendicular to the gold electrodes of the substrate.

**Template-Assisted Self-Assembly of AuNRs.** The substrates were cleaned by sonication in acetone and isopropanol, consecutively, blow-dried (air) and cleaned for 5 min in UV/ozone. The AuNRs were assembled from the aqueous solution. Of a 10 mg/mL solution with 1 mM CTAC, 2 µL was deposited on top of a substrate and a 12 × 12 mm PDMS template with the wrinkles being oriented perpendicular to the gold electrodes (Figure 2a) was left on top for 4 h, giving rise to the AuNR lines due to confinement assembly (see Figure 1c). Usually, a AuNR line has a width of 300 nm and is formed by the template with a periodicity of around 905 nm, see Figure 2a.

**Electrical Measurements**. Electrical measurements were executed at room temperature under nitrogen in a Lake Shore Cryotronics probe station CRX-6.5K with a Keithley 2634B System Source Meter, and the samples were contacted with tungsten two-point probes.

**Scanning Electron Microscopy**. Scanning electron micrographs were taken using a HITACHI model SU8030 at 30 kV.

## 3. Results and Discussion

### 3.1. Fabrication

Gold nanorods (AuNRs) with an aspect ratio of 7.3 were synthesized as previously described. Shortly, seeded growth was performed with tetrachloroauric acid in aqueous solution of Cetyltrimethylammoniumbromide (CTAB) with AgNO_3_ and hydroquinone. Into this mixture, single crystalline gold seeds were injected rapidly while vortexing by hand, followed by overnight resting at 32 °C, exchange of the stabilizing surfactant to CTAC, and purification. Thorough purification and consistent behavior of the colloids would not have been ensured with CTAB due to crystallization [34]. The exchange of the surfactant from CTAB to CTAC, however, enabled easy handling of the colloidal AuNRs at room temperature.

The positions of the plasmonic modes of the AuNRs in the UV-vis spectrum (Figure 1a) at 505 nm (transversal) and at 1107 nm (longitudinal) correlate to the length of 118 ± 16 nm and width of 16 ± 1 nm derived from the TEM images (Figure 1b and Figure S1) [35,36].

To create AuNR lines, template-assisted self-assembly with wrinkled PDMS templates was used, since it allowed for easy and cost-effective assemblies on various substrates [37,38]. We chose an FET substrate consisting of a Si/SiO_2_(230 nm) wafer with photolithographically deposited gold electrodes. The PDMS templates were fabricated by treating stretched PDMS stripes with oxygen plasma, followed by relaxation. This results in PDMS stripes with a wrinkled surface of sinusoidal shape. By changing the parameters of the plasma treatment, the geometrical dimensions of the wrinkles, periodicity and feature height can be tuned [39,40,41] and thereby, adjusted exactly to the required dimensions of the attempted application. AuNRs were assembled from aqueous solution into lines by confinement assembly (see Figure 1c). This is a template-assisted self-assembly method in which the colloids are confined between the PDMS template and the substrate [37]. The AuNR lines formed in the grooves of the wrinkles (Figure 1c). The PDMS templates had a periodicity of 950 nm and depth of 220 nm. These dimensions allowed for the flow of the AuNRs through the channels between the substrate and the template during the assembly process, but still provided enough confinement to result in narrow AuNR lines, with widths of 319 ± 139 nm (Figure 2a).

### 3.2. Measurement Results

To measure the resistance of these AuNR lines, the electrode array of the substrate with parallel gold electrodes was used. The AuNR lines were assembled perpendicular to the electrodes, thereby connecting the electrodes (see Figure 2b). The center-to-center distance of the AuNR lines was about 905 ± 31 nm. The resistance was measured between each of the two parallel gold electrodes with a distance (channel length) of 1.5 µm.

**Figure 3:**
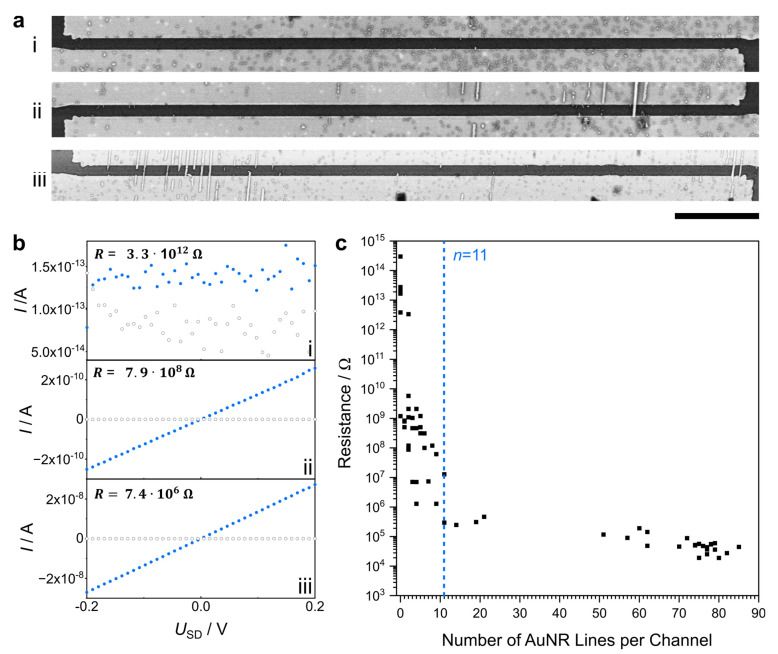
(**a**) SEM images, scale bar: 10 µm, and (**b**) the corresponding *I–U* plots obtained from source-drain measurements of the shown channels without a continuous AuNR line. (i) One AuNR line (ii) and six AuNR lines (iii), with blue = source-drain current, grey = leakage current, and (**c**) the plotted resistances for all channels.

The measured resistances were then correlated with SEM images of the AuNR lines. For each measured channel, the resistance and total number of continuous, electrode-connecting AuNR lines was noted as in the examples in Figure 3a,b. Any AuNR lines with gaps larger than 50 nm in between were not counted. This “coarse-grained” approach still does not rule out that there is no conductivity due to smaller gaps, as will become apparent in further discussion. As electronic conductivity decreases exponentially with the increasing spacing between the gold NPs [42,43,44,45,46], we do not expect charge to be transferred from one AuNR to another if the gap between them is 5–7 nm or larger [26].

The channel in Figure 3 (i) does not show a continuous AuNR line, and consequently, there is no conductance (R = 3.3 TΩ). In Figure 3 (ii) there is one continuous AuNR line visible and, thus, to that, the resistance measurement shows a much lower resistance of 79 GΩ. This is comparable with the resistances of 1 nm spaced gold nanowires, which have similar widths as this AuNR line [47]. In accordance with the literature of colloidal nanowires, the AuNR line shows an ohmic behavior at room temperature in this low-voltage regime [48,49]. The channel depicted in Figure 3 (iii) is connected by six AuNR lines, and a resistance of 7.4 MΩ was measured. For all the studied channels with a conductivity higher than the detection limit, the current-vs-voltage curves exhibited ohmic behavior, independent of the number of connecting AuNR lines. After acquiring the data for all channels, we correlated the number of apparently continuous AuNR lines with the corresponding resistances (see Figure 3c).

### 3.3. Discussion

In Figure 3c, the measured resistances for each channel are plotted against the corresponding number of continuous AuNR lines, *n*. The more AuNR lines connect a pair of electrodes, the lower the measured resistance. The large scattering of the resistances for a lower *n* can be attributed to AuNR lines with strongly deviating resistance due to lower or a complete lack of conductance. If a channel has only a few conductive AuNR lines, its conductivity will be more severely affected by a low or non-conductive line, as is the case with many lines per channel. Unlike the scattering resistances for low numbers of AuNR lines, the total channel resistances converge for *n* > 11 and are constant within one order of magnitude (~10^5^ Ω). Therefore, it can be concluded that more than 11 AuNR lines are needed for reliable conductance.

As for the non-conductive AuNR lines, we cannot rule out that some seemingly channel-bridging AuNRs still have charge-transport interrupting gaps which could not be detected by the coarse-grained SEM method. For the conducting AuNR lines, the reasons for deviating conductance can be manifold. Firstly, they can be attributed to the nonuniform arrangement of AuNRs in different AuNR lines. This causes a distribution of the number of charge–transport paths in the AuNR lines (corresponding to resistors connected in parallel) and of the number of resistive gaps within such a charge–transport path (corresponding to resistors connected in a series). Secondly, as we work with a CTAC concentration around the critical micelle concentration during the AuNR line assembly, the formation of the CTAC bilayers around the AuNRs could differ from one AuNR to another. The formation of the CTAC bilayers between the AuNRs is linked to the gap size and the latter one to the resistance of this gap. Assembled CTAC-stabilized AuNRs were shown to have a minimum distance of 3.4 nm, which corresponds to the thickness of a shared interdigitated CTA^+^ bilayer [50]. The thickness of CTA^+^ multilayers can also be significantly larger (i.e., 9 nm) as Sau and Murphy [50] reported, thus giving rise to a larger gap resistance, contributing to an increased total resistance of the AuNR line.

To attempt a more detailed account of the consistency of the resistances, we consider the AuNR lines as ohmic resistors $R_{i}$ connected in parallel. The measured total resistance $R_{\mathrm{total}}$ of each channel then results as:

(1) $$\;\;\;\frac{1}{R_{\mathrm{total}}}=\sum_{i=1}^{n}\frac{1}{R_{i}},$$

with $R_{i}$ being the resistance of an individual AuNR line in one channel. We cannot know the resistance of every single AuNR line, but we can model the resistance with the assumption that every AuNR line in a considered channel has the same resistance, $R_{\mathrm{single}}$. Although this assumption may have weaknesses (as can be seen from the comparably large scattering of resistances in Figure 3c), it helps in assessing the resistance measurement data. Assuming all $R_{i}$ = $R_{\mathrm{single}}$, Equation (1) yields

(2) $$\frac{1}{R_{\mathrm{total}\;}}=n\cdot \frac{1}{R_{\mathrm{single}}},$$

with *n* as the total number of continuous AuNR lines within the relevant channel. Figure S2 shows that the $R_{\mathrm{single}}$ values are roughly constant for *n* > 11, and therefore supports the assumption of our model of uniform AuNR lines acting as ohmic resistors connected in parallel. To calculate a general $R_{\mathrm{single}}$, we can use a linear regression for the total channel resistances, $R_{\mathrm{total}}$ versus $\frac{1}{n}$, with $R_{\mathrm{single},\;\mathrm{fitted}}$ as the slope of the linear graph:

(3) $$R_{\mathrm{total}}=R_{\mathrm{single},\;\mathrm{fitted}}(n>11)\cdot \frac{1}{n}.$$

**Figure 4:**
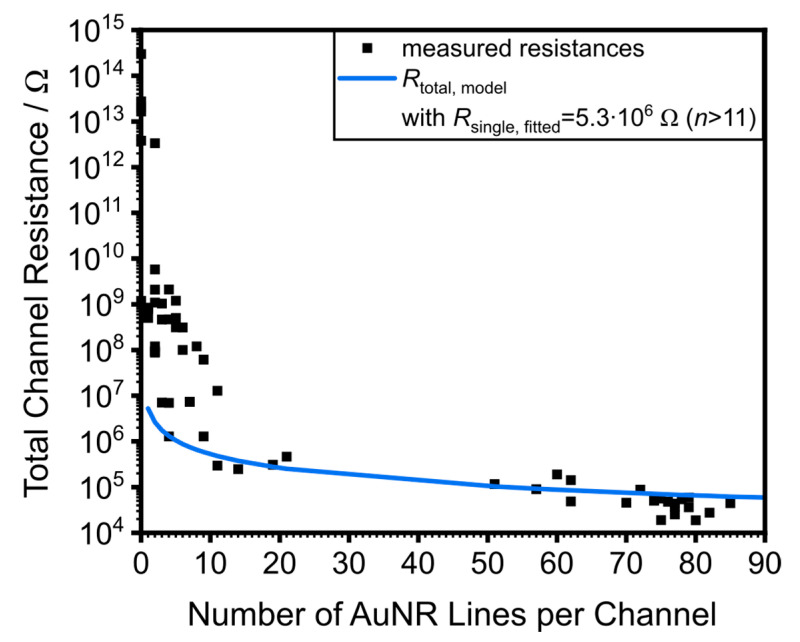
Conductivity measurements: measured and modeled total channel resistances.

With a coefficient of determination of 0.81, this linear regression gives a slope, i.e., $R_{\mathrm{single},\;\mathrm{fitted}}$ of $5.3\cdot 10^{6}$ Ω (see Figure S3). This single AuNR line resistance is in the range of the lowest resistances measured for similarly sized gold nanowires, which were about 1 nm apart from each other [47]. This shows that despite the surfactant-induced gaps, the charge transport along the AuNR lines works remarkably well. Inserted into the plot of the total channel resistances versus the number of AuNR lines per channel (Figure 4), the modeled total channel resistance, $R_{\mathrm{total},\mathrm{model}}$, fits the measured data well for the ohmic-resistors regime for *n* > 11 and even for the smallest values of the total channel resistances for a lower *n*.

Our conductivity measurement results mirror the heterogeneities of the AuNR arrangement between different AuNR lines, which results in strongly fluctuating resistances for small numbers of AuNR lines. However, by connecting electrodes with several (*n* > 11) AuNR lines, this heterogeneity does not negatively impair the consistency of conductivity measurements. Hence, the assembly of multiple conductive supracolloidal lines offers a suited approach to mitigate inconsistency in the transport behavior of this promising class of mesoscale electronic materials. This approach takes up an area of 10.8 µm × 1.5 µm for 12 parallelly aligned AuNR lines with a center-to-center distance of about 950 nm. Such a system still shows higher conductance (~10^−5^ S) than a monolayer of gold nanospheres with dithiolated-conjugated ligands (>10^−7^ S) [31], and additionally features the advantage of anisotropic conductance.

## 4. Conclusions

In summary, we successfully fabricated AuNR lines via template-assisted self-assembly and characterized their conductance. By using bottom-up fabricated PDMS templates and wet-chemically synthesized AuNRs for the confinement assembly, the whole process of the linear assembly did not require expensive equipment. Another advantage is the possibility to print these AuNR lines on a plethora of materials [37,38], including heat-sensitive polymer films, since our fabrication process does not include sintering. Additionally, our structures feature comparably low dimensions in terms of the AuNR line width [3,30]. We observed a dependence of the conductance on the number of channel-bridging AuNR lines. For more than 11 AuNRs per channel, the single-line resistances approached a unified behavior, described by the ohmic model of uniform resistors connected in parallel. The results demonstrate that consistent conductivity properties can be reached if several supracolloidal wires are employed, even if their conductivity properties fluctuate strongly among the individual lines. This is especially applicable to the development of sensors based on surfaces with anisotropic resistance properties. With our approach, the active areas can be as small as 16 µm^2^, but also as large as cm^2^ [18], depending on the intended application. In this regard, the up-scalable fabrication and integration of our AuNR lines into robust technical processes is promising for future device integration [51].

## Data Availability

The data presented in this study are available on request from the corresponding authors.

## Supplementary Materials

The supporting information can be downloaded at: https://www.mdpi.com/article/10.3390/nano13091466/s1, Figure S1: Histograms of the AuNRs’ dimensions, their width and length, derived from TEM measurements; Figure S2: Conductivity measurements: Calculated mean resistance for single AuNR lines; Figure S3: Linear fit of the measured R_total and model for R_total by using the R_(single, fitted) derived from the linear regression; Figure S4: Conductivity measurements: (a) calculated conductances per AuNR line and (b) total channel conductances G, measured and modeled values; Figure S5: The measured channel resistances for *n* = 1, *n* = 2, *n* = 3 and *n* = 4 exemplarily illustrate the scattering of the total channel resistances R_total for small *n*.
