# Supplementary material for: Heading toward Miniature Sensors: Electrical Conductance of Linearly Assembled Gold Nanorods
Source: Nanomaterials (Basel). 2023 Apr 25;13(9):1466. doi: 10.3390/nano13091466 (PMC10179793; doi:10.3390/nano13091466)
Supplement: Supplementary file 1 [file nanomaterials-13-01466-s001.zip › nanomaterials-2341493-supplementary.pdf]

Supporting Information to

Heading Toward Miniature Sensors: Electrical Conductance of Linearly Assembled Gold Nanorods

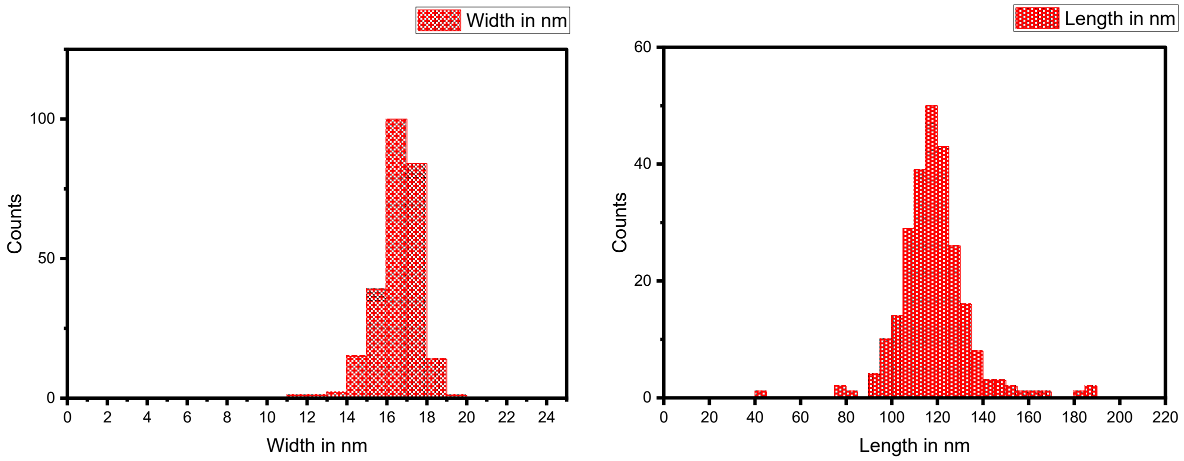

Figure S1: Histograms of the AuNRs' dimensions, their width (left) and length (right), derived from TEM measurements.

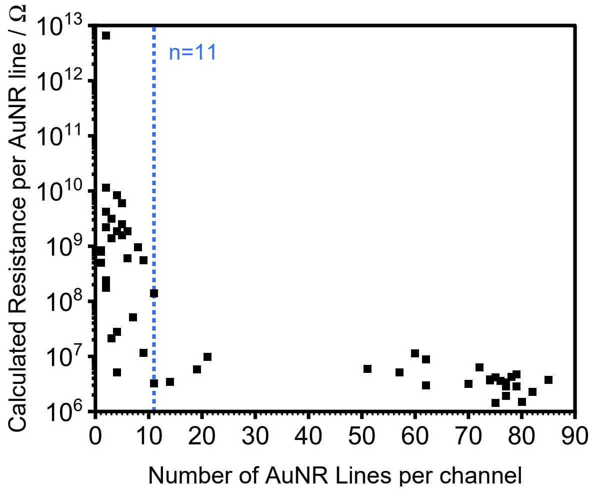

Figure S2: Conductivity measurements: Calculated mean resistance for single AuNR lines, R_{single}.

A mean resistance of a single AuNR line in a certain channel, $R_{\text{single}} = n \cdot R_{\text{total}}$ can be calculated, corresponding to AuNR lines acting as resistors with uniform resistance connected in parallel. If this assumption is correct, the calculated resistances per AuNR line, R_{single} should be constant over all numbers n of AuNRs. However, as can be seen from the figure, the more AuNR lines connect a pair of electrodes, the lower is R_{single}. Only for more than 11 AuNR lines per channel, the R_{single} values are more or less constant and therefore, meet the

assumption of our model of uniform AuNR lines acting as ohmic resistors connected in parallel.

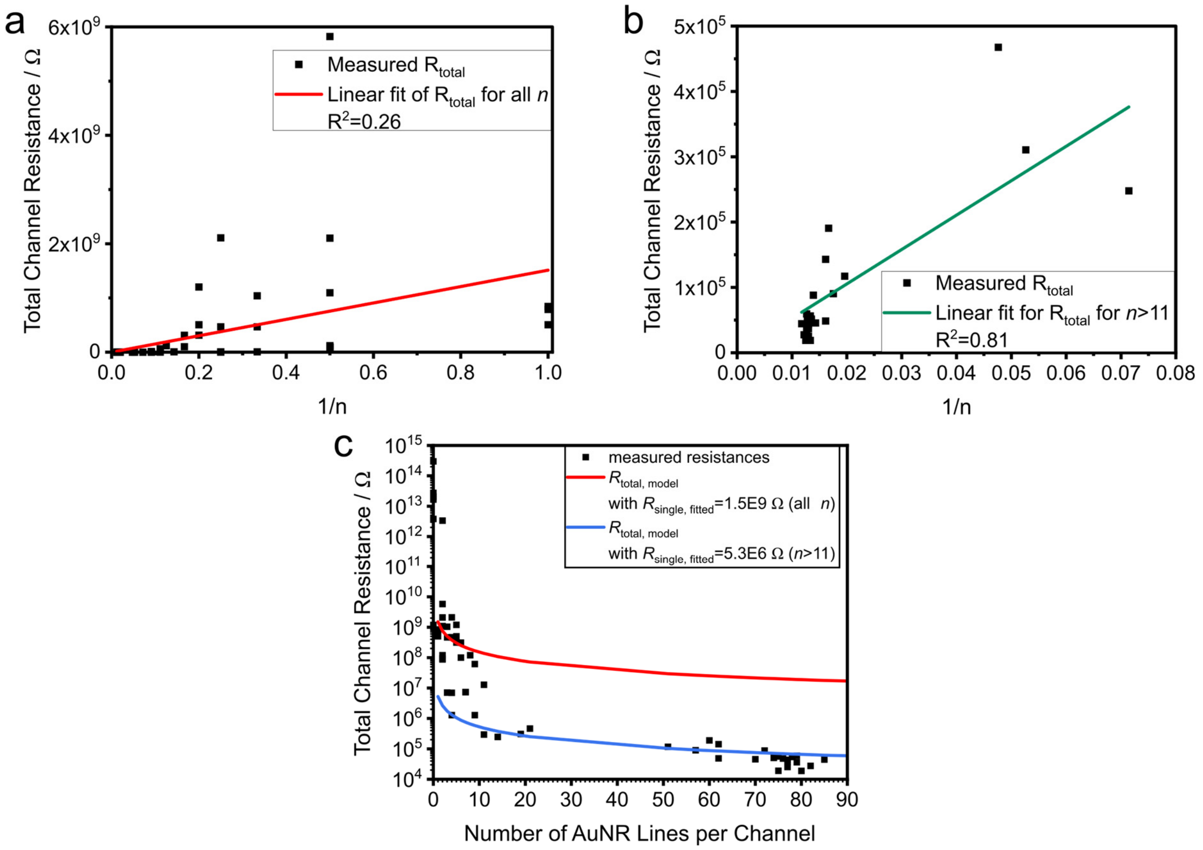

Figure S3: Linear fit of the measured R_{total}, including (a, red) all total channel resistance values except one outlier R_{total}=3.35 TΩ, for n=2 and (b, green) only the total channel resistance values for n>11, respectively. (c) All measured total channel resistances and the model for R_{total} by using the $R_{\text{single,fitted}}$ derived from the linear regression.

If all measured R_{total} for 1≤n≤85 are used to calculate R_{single} by regression with the equation

$$R_{\text{total,mean}} = R_{\text{single,fitted}} \cdot \frac{1}{n}$$

the linear regression unsurprisingly is not satisfactory and has a very low coefficient of determination (R^2) of 0.26, even if we omit the clear outlier R_{total}=3.35 TΩ for n=2. Hence, the corresponding model for R_{total} does not fit the experimental data well (c, red graph). The overall trend of the measured R_{total} cannot be reproduced, as for n≤11 the measured resistances do not provide an adequate base to calculate a model $R_{\text{total,model}}$. On the contrary, the model $R_{\text{total,model}}(n \geq 11)$ fits the data well for the ohmic-resistors-regime (c, blue graph).

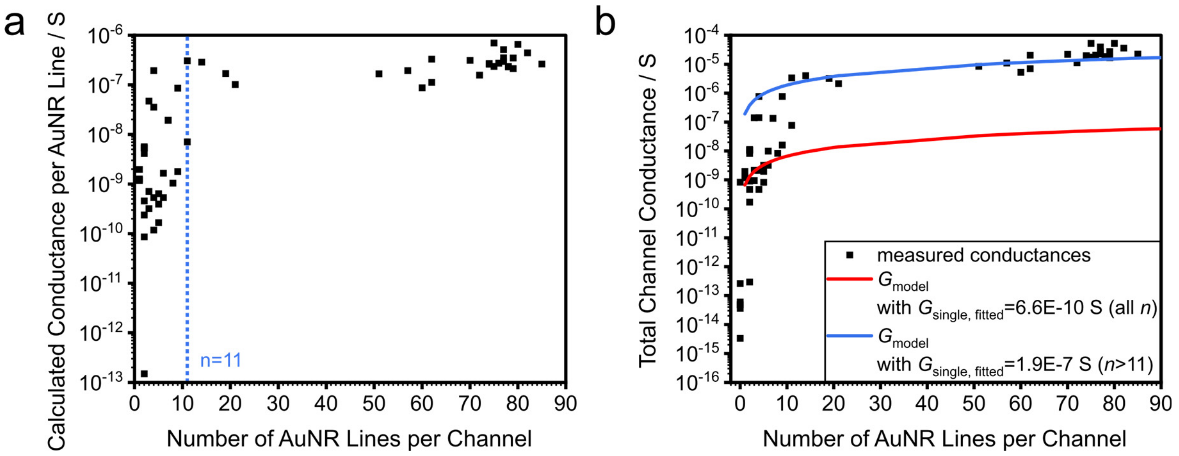

Figure S4: Conductivity measurements: (a) calculated conductances per AuNR line and (b) total channel conductances G, measured and modeled values.

The equation used for a) is $G_{\text{total}} = n \cdot G_{\text{single,fitted}}$.

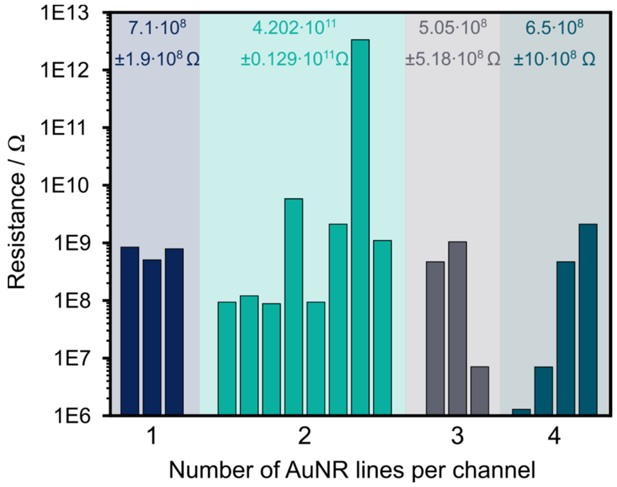

Figure S5: The measured channel resistances for n=1, n=2, n= 3 and n=4 exemplarily illustrate the scattering of the total channel resistances R_{total} for small n.
